# Berbamine exerts anti-breast-cancer effects in association with autophagy-related responses and PI3K/Akt1/mTOR signaling

**DOI:** 10.3389/fphar.2026.1976563

**Published:** 2026-10-29

**Authors:** Rui Ma, Lingchen Liao, Gang Fang, Jinlin Zheng, Ziwei Xia, Shuqi Chen, Yuzhou Pang, Zhiyong Cao

**Affiliations:** 1 Traditional Therapy Department, Guangdong Provincial Hospital of Chinese Medicine, Guangzhou, China; 2 Guangxi Higher Education Key Laboratory for the Research of Du-related Diseases in Zhuang Medicine, Guangxi University of Chinese Medicine, Nanning, China; 3 Institute of Traditional Chinese and Zhuang-Yao Ethnic Medicine, Guangxi University of Chinese Medicine, Nanning, China; 4 Acupuncture Department, Guangdong Provincial Hospital of Chinese Medicine, Guangzhou, China

**Keywords:** antitumor activity, autophagy, berbamine, breast cancer, PI3K/AKT/mTOR

## Abstract

**Introduction:**

Breast cancer remains a leading cause of cancer-related morbidity and mortality worldwide, and effective, well-tolerated therapeutic strategies are needed. Berbamine (BBM), a natural bisbenzylisoquinoline alkaloid, has anticancer activity, but its effects on autophagy- and lysosome-related processes in breast cancer remain incompletely understood.

**Methods:**

We evaluated the effects of BBM on MDA-MB-231, MDA-MB-468, MCF-7, and 4T1 breast cancer cells and normal mammary epithelial cells using assays of cell viability, migration, invasion, autophagy-related responses, lysosome-associated changes, immunofluorescence, transmission electron microscopy, and Western blotting. Exploratory network analysis was used to prioritize candidate BBM-associated pathways, and molecular docking was used to predict interactions between BBM and selected pathway proteins. Antitumor activity was further assessed in an MCF-7 xenograft model.

**Results:**

BBM inhibited the proliferation, migration, and invasion of breast cancer cells while showing relatively low cytotoxicity toward normal mammary epithelial cells. BBM treatment was associated with increased levels of p-ULK1, ATG5, ATG7, and Beclin1, increased LC3 puncta, and accumulation of double-membraned autophagosomes. BBM was also associated with TFEB nuclear translocation, increased LAMP2 expression, and enhanced LysoTracker staining. Network analysis prioritized the PI3K/Akt signaling pathway, and molecular docking predicted potential interactions between BBM and PI3K, Akt1, and mTOR. Western blot analysis showed reduced phosphorylation of these proteins in cultured breast cancer cells. In the MCF-7 xenograft model, BBM inhibited tumor growth and was associated with changes in autophagy-related markers.

**Discussion:**

These findings support the involvement of autophagy-related responses, lysosome-associated changes, and PI3K/Akt1/mTOR pathway modulation in the anti-breast-cancer effects of BBM, while further studies are needed to establish direct target engagement and causal relationships.

## 1 Introduction

Breast cancer remains the most commonly diagnosed malignancy among women worldwide and is a leading cause of cancer-related mortality (Terranova-Barberio et al., 2020). According to the Global Cancer Statistics 2022 published by the International Agency for Research on Cancer (IARC), breast cancer accounted for approximately 2.31 million new cases in the world and ranked second in incidence and fourth in mortality among all cancers. In China, breast cancer comprised an estimated 357,000 new cases in 2022, which accounted for 15.5% of global cases and ranked second among female cancers in incidence (Bray et al., 2024). Although early detection and advances in treatment have improved outcomes, the disease burden remains high, particularly among women in both developed and developing countries (Bray et al., 2024).

Autophagy is an evolutionarily conserved catabolic process responsible for the degradation and recycling of damaged organelles and misfolded proteins, particularly under stress conditions (Wang et al., 2020; Lee et al., 2022; Niu et al., 2023). Autophagy functions as a tumor suppressor in early stages and a survival mechanism in established tumors, indicating a complex dual role in cancer (Yu et al., 2024). The dysregulation of autophagy has been implicated in various cancers (Niu et al., 2025), including breast cancer (Hashemi et al., 2023), where it influences tumor progression, metastasis, and therapeutic resistance (Elshazly and Gewirtz, 2023). Pro-survival autophagy can reduce the efficacy of anticancer therapies, but excessive or dysregulated autophagy can lead to autophagic cell death (Liu et al., 2024; Wen et al., 2024), thus offering a promising avenue for therapeutic intervention.

Recent studies have highlighted the importance of modulating autophagy in breast cancer treatment (Niklaus et al., 2021; Kadkhoda et al., 2022; Wu and Sharma, 2023). Key signaling pathways, such as the PI3K/Akt/mTOR cascade, tightly regulate autophagy and are frequently dysregulated in breast cancer. The inhibition of this pathway has been shown to promote autophagy and suppress tumor progression (Cocco et al., 2022). For instance, BMP9 was reported to suppress breast cancer metastasis by inducing autophagy through the c-Myc/SNHG3/mTOR axis, indicating the therapeutic potential of targeting this pathway (Yu et al., 2023).

Berbamine (BBM), a natural bisbenzylisoquinoline alkaloid extracted from traditional Chinese medicinal herbs, has attracted considerable interest due to its broad pharmacological activities, including anti-inflammatory, antioxidant, and anticancer properties (Liu et al., 2023). Unlike many synthetic chemotherapeutics, BBM also exhibited low toxicity in normal cells, making it a promising candidate for anticancer therapy. BBM has demonstrated anti-proliferative and pro-apoptotic effects in various cancers, including colon, liver, and breast cancers (Liu et al., 2010; Peng et al., 2024; Muhammad et al., 2025). Moreover, BBM has been reported to inhibit the PI3K/Akt/mTOR signaling pathway (Liu et al., 2021), modulate key autophagy-related regulators such as BNIP3, SNAP29, and VAMP8, and impair autophagosome-lysosome fusion, thereby inducing autophagic stress and cell death (Fu et al., 2018). Moreover, BBM-mediated autophagy has shown benefits in other diseases such as non-alcoholic fatty liver disease and neurodegeneration (Sharma et al., 2021; Faheem et al., 2022), further supporting its pleiotropic regulatory role.

This study systematically evaluated the anti-breast-cancer effects of BBM and examined the associated autophagy-, lysosome-, and signaling-related changes. To identify pathways for experimental evaluation, we combined exploratory network analysis with evidence from previous studies of autophagy and BBM in breast cancer. We prioritized the PI3K/Akt1/mTOR pathway for experimental evaluation because it is an established regulator of autophagy and cell survival, is frequently dysregulated in breast cancer, and has been implicated in BBM-related responses in previous studies. Western blot analyses showed reduced phosphorylation of PI3K, Akt1, and mTOR in cultured breast cancer cells, while reduced p-mTOR was observed in xenograft tumors. These findings support an association between BBM treatment and PI3K/Akt1/mTOR pathway modulation and provide a rationale for further mechanistic investigation.

## 2 Methods

### 2.1 Cell culture

MCF-7, MDA-MB-231, MDA-MB-468, and 4T1 were cultured in DMEM supplemented with 10% fetal bovine serum (Sigma, F0193-500 mL), 100 U/mL penicillin, and 10 μg/mL streptomycin. MCF-10A cells were cultured in MCF-10A Cell Complete Medium (Procell, CM-0525). Cells were maintained at 37 °C in a humidified incubator with 5% CO_2_. The culture medium was refreshed daily, and cells were subcultured at 80% confluence.

### 2.2 Reagents

LysoTracker Red DND 99 (HY-D1300), Berbamine (HY-N0714), CCK-8 (HY-K0301), Hoechst 33,342(HY-15559), Bafilomycin A1 (HY-100558) and Rapamycin (HY-10219) were obtained from MedChemExpress. mRFP-GFP-LC3 assay were obtained from HANBIO.

### 2.3 Cell proliferation assay

Cell inhibition was monitored by CCK-8 assay. Cells were seeded in 96-well plates. After the treatment, the culture medium was removed. Subsequently, CCK-8 was added into each well and incubated for 2.5 h after the removal of supernatants. The absorbance was recorded at 450 nm under a microplate reader (Bio-Tek Instruments, Highland Park, Winooski, VT, United States). Cell inhibition rate is calculated as:

$$\text{Inhibition rate }\left(\%\right)=\left[\left(A_{450},\;\text{Control}-A_{450},\;\text{Sample}\right)\;/\;\left(A_{450},\;\text{Control}-A_{450},\;\text{Blank}\right)\right]\times 100\%.$$

### 2.4 Use of autophagy inhibitors and activators

MCF-7 and MDA-MB-231 cells were treated with BBM alone or in combination with rapamycin (Rapa) or bafilomycin A1 (Baf-A1). BBM was used at 25 μM, rapamycin at 250 nM, and Baf-A1 at 20 nM for 24 h. Control cells received the corresponding vehicle. Cell viability was assessed using the CCK-8 assay. For Western blot analysis, cells were harvested after treatment, and cleaved caspase-3 expression was analyzed as described in Section 2.11. Each experiment was performed in three independent replicates.

### 2.5 Cell invasion assay

Matrigel was diluted with serum-free medium and added to the upper chamber of the Transwell insert. The inserts were incubated at 37 °C for 1–2 h to solidify the Matrigel. Cells were digested, centrifuged, and resuspended in serum-free medium at a density of approximately 1 × 10^5^ cells/mL. The cell suspension was added to the upper chamber, and the lower chamber was filled with medium containing serum as a chemoattractant. The Transwell chambers were placed into a 24-well plate and incubated at 37 °C in a 5% CO_2_ incubator for 48 h. After the incubation, the chambers were removed and washed with PBS. Cells were fixed with 4% paraformaldehyde for 30 min, followed by staining with 0.1% crystal violet for 20 min. Non-invading cells on the upper surface of the membrane were carefully removed with a cotton swab. Cells that had invaded to the lower surface were observed and counted under a microscope.

### 2.6 Wound healing assay

Cells were seeded in 6-well plates and cultured until they reached 90%–100% confluence. A sterile pipette tip or wound scratcher was used to generate a straight scratch across the cell monolayer. Detached cells were removed with PBS. Serum-free medium was then added, and the plates were incubated at 37 °C in a 5% CO_2_ incubator. At 48 h post-scratch, wound closure was observed and photographed under a microscope. The scratch width was measured in ImageJ, and the cell migration rate was calculated accordingly.

### 2.7 Experimental animals

A total of 20 BALB/c nude mice (4–6 weeks) were obtained and housed in the SPF-grade barrier facility of the Key Laboratory at the Guangxi University of Chinese Medicine. The mice were provided with sterilized commercial chow and autoclaved drinking water, with *ad libitum* access to food and water. Sterile chow and water were replaced daily, and bedding was changed every 2 days to ensure a hygienic environment. After a 7-day acclimatization period, five female BALB/c nude mice were randomly selected as the normal control group, whereas the remaining mice were used for model establishment. All animal procedures were conducted in accordance with the ethical guidelines of Guangxi University of Chinese Medicine (Approval No. DW20241025-01).

### 2.8 Establishment of a breast cancer xenograft model

After wearing sterile gloves, each nude mouse was restrained by firmly grasping the skin on the back of the neck with the thumb, index, and middle fingers, while the base of the ring and little fingers stabilized the tail. The mouse was positioned head-down with the right lateral side facing inward to expose the right forelimb. The axillary region of the right forelimb was disinfected with an alcohol swab. With a 1-mL syringe, 0.1 mL of the suspension of human breast cancer MCF-7 cells (1 × 10^7^ cells/mL) was aspirated and injected subcutaneously approximately 1 cm below the right axilla at a depth of ∼1 cm. After injection, the site was pressed with an alcohol swab for 10–30 s to prevent the leakage of the cell suspension.

### 2.9 Model validation and grouping

During the modeling period, tumor growth in nude mice was closely monitored and recorded. Tumor size was measured with a Vernier caliper. Successful tumor formation was defined as the presence of a palpable or visibly detectable mass, with a tumor volume ≥150 mm^3^, as determined by caliper measurements. Mice meeting this criterion were considered to be successfully modeled and included in the treatment groups. After 21 days, only twelve mice met the inclusion criteria and were randomly assigned to two groups: the model control group and the BBM treatment group, with six mice each.

### 2.10 Drug administration and tissue collection

The treatment group received BBM (100 mg/kg, intragastric administration) (Meng et al., 2013), while the control group received sterile water (5 mL/kg, intragastric administration). BBM was administered once daily for 14 consecutive days. Body weight and tumor volume were recorded every 2 days. After the 14-day treatment period, all mice were euthanized by cervical dislocation. Tumor tissues were harvested for subsequent pathological and experimental analyses.

### 2.11 Histopathology

Tumor tissues were fixed in 4% paraformaldehyde for 7 days. Then, tissues were embedded in paraffin, sectioned, dewaxed in xylene, and rehydrated through graded ethanol. Images were observed with an Olympus BX53M microscope, and quantitative analysis was performed using ImageJ.

### 2.12 Western blot

Protein expression in the cell lines were investigated by Western blot according to our previous method (Sun et al., 2025). Antibodies used for western blots include anti-LC3 (Cell Signaling Technology, 3868S), anti-p62/SQSTM1 (Santa Cruz Biotechnology, 48,402), anti-ATG5 (Santa Cruz Biotechnology, 133,158), anti-ATG7 (Santa Cruz Biotechnology, 376,212), anti-p-PI3K (Cell Signaling Technology, 4249), anti-PI3K (Cell Signaling Technology, 4263), anti-p-AKT1 (Cell Signaling Technology, 13,038), anti-AKT1 (Cell Signaling Technology, 2938), anti-p-mTOR Ser2448 (Cell Signaling Technology, 5536S), anti-mTOR (Cell Signaling Technology, 2983S), anti-p-ULK1 Ser555 (Cell Signaling Technology, 5,869), anti-Beclin1 (Santa Cruz Biotechnology, 48,381), anti-TFEB (Proteintech, 13,372), anti-Caspase-3 (Cell Signaling Technology, 9,662), anti-Cleaved Caspase-3 (Cell Signaling Technology, 9,661), and anti-β-actin (Cell Signaling Technology, 4970S).

### 2.13 mRFP-GFP-LC3 assay

To thaw the virus, it was melted on ice. For dilution, the initial virus titer was calculated to be 1 × 10^10^ PFU/mL based on the original virus labeling. For example, mixing 10 µL of virus with 990 µL of standard culture medium resulted in a virus titer of 1 × 10^8^ PFU/mL. The appropriate MOI (multiplicity of infection) was selected based on the cell growth rate.

MCF-7 or MDA-MB-231 cells were digested and diluted to a concentration of 6 × 10^4^ cells/mL. Then, 100 µL of the diluted medium containing 6 × 10^3^ cells was added per well in a 96-well plate and incubated in a 5% CO_2_ incubator at 37 °C for 24 h.

For adenovirus infection, half of the volume of adenovirus was added initially and incubated for 4 h. After 4 h, the remaining volume was added to reach the total culture volume (100 µL for the 96-well plate or 1 mL for confocal dishes). Cells were further incubated for 8 h. Then, the virus-containing medium was removed, replaced with fresh complete culture medium, and returned to a 5% CO_2_ incubator at 37 °C for continuous cultivation.

After 12 h of complete culture, the supernatant was removed, and each well was supplemented with 100 µL of complete culture medium containing BBM. Cells were incubated at 37 °C. After 48 h of drug treatment, fluorescent signals were observed with the Operetta PerkinElmer Opera Phenix High Content Screening System.

### 2.14 LysoTracker red DND 99 assay

LysoTracker Red DND 99 working solution was prepared as follows. Firstly, the solution temperature was restored to room temperature. After centrifugation, cells were concentrated at the bottom of the tube. Then, the stock solution (1 mM) was diluted with culture medium or an appropriate buffer, such as PBS, to prepare the working solution (100 nM). Cell staining was performed as follows. For adherent cells, after the cell culture medium was discarded, cells were dissociated with trypsin to prepare a single-cell suspension. After centrifugation for 3–5 min (4 °C, 1000 g), the supernatant was discarded. Then, cells were washed twice with PBS for 5 min. After 100 μL of LysoTracker Red DND 99 working solution was added, cells were incubated at room temperature for 5–30 min. After centrifugation (4 °C, 400 g) for 3–4 min, the supernatant was discarded. Then, cells were washed twice with PBS for 5 min. Cells were resuspended in serum-free cell culture medium or PBS and analyzed with the Operetta CLS High Content Screening Imaging System.

### 2.15 Immunofluorescence assay

For immunofluorescent colocalization analysis, MCF-7 and MDA-MB-231 cells were cultured in confocal dishes (Corning) for 24 h and treated with 25 µM BBM for 48 h. Cells were fixed and incubated with anti-TFEB overnight at 4 °C. After incubation with secondary antibodies and Hoechst 33,342, confocal imaging was performed using the Operetta PerkinElmer Opera Phenix High Content Screening System.

### 2.16 Exploratory network analysis of candidate BBM-associated targets and pathways

Disease-related targets associated with breast cancer were obtained from the Gene Expression Omnibus (GEO) database (https://www.ncbi.nlm.nih.gov/geo/). Two eligible datasets, GSE42568 and GSE124646, were selected based on the following criteria: the samples were derived from human breast cancer and adjacent normal tissues, and the datasets contained at least 10 samples per group. Differentially expressed genes (DEGs) between breast cancer and normal tissues were identified using the GEO2R tool (https://www.ncbi.nlm.nih.gov/geo/geo2r/).Genes were considered differentially expressed according to an adjusted p-value <0.05 and an absolute log2 fold-change ≥1. The DEG lists from GSE42568 and GSE124646 were analyzed separately and then combined to define the union of breast-cancer-associated DEGs for exploratory target-overlap analysis.

The chemical structure of berbamine was obtained from the PubChem database (https://pubchem.ncbi.nlm.nih.gov/). Putative targets of berbamine were predicted using the SwissTargetPrediction platform (http://www.swisstargetprediction.ch/), with the species set to *Homo sapiens* and a probability threshold >0.

The overlap between the breast-cancer-associated DEGs and the predicted BBM targets was identified using the Venn diagram tool. The resulting 55 genes were treated as candidate BBM-associated targets for hypothesis generation. Because these targets were inferred computationally, they were not considered experimentally validated drug targets. The relationships among BBM, breast cancer, and the candidate targets were visualized using Cytoscape software. (version 3.9.1; https://cytoscape.org/).

For exploratory functional annotation, the 55 candidate BBM-associated targets were subjected to enrichment analysis using the Database for Annotation, Visualization and Integrated Discovery (DAVID) v6.8 (https://david.ncifcrf.gov/). The enrichment results were used to nominate candidate pathways for further investigation. Kyoto Encyclopedia of Genes and Genomes (KEGG) pathway enrichment and Gene Ontology (GO) biological process analyses were performed. The top 20 KEGG pathways and GO terms ranked by gene count were visualized using the bioinformatics online platform (https://www.bioinformatics.com.cn/).

### 2.17 Molecular docking

The molecular docking analysis was performed to predict the potential interaction between BBM and key proteins involved in the PI3K/Akt1/mTOR signaling pathway. The three-dimensional structures of target proteins (PI3K (PDB ID: 1E8Z), Akt1 (PDB ID: 3O96), and mTOR (PDB ID: 8PPZ)) were obtained from the RCSB Protein Data Bank. The 3D structure of BBM was downloaded from the PubChem database and energy-minimized with Chem3D software.

All protein structures were preprocessed by removing water molecules and ligands, adding hydrogen atoms, and assigning Kollman charges with AutoDock Tools. Docking simulations were conducted with AutoDock Vina. Grid boxes were set to cover the active sites of each protein based on previously reported key residues. Docking results were evaluated based on binding affinity (kcal/mol) and binding modes. The best-scored conformations were visualized and analyzed with PyMOL and Discovery Studio Visualizer to identify potential hydrogen bonds, hydrophobic interactions, and π-π stacking between BBM and the target proteins.

### 2.18 Statistical analysis

Statistical analyses were conducted with SPSS version 26.0 and GraphPad Prism Version 9 software. Experimental data were expressed as mean ± SEM. Comparisons between two groups were analyzed through Student’s t-test, whereas the comparisons among multiple groups were conducted through ANOVA and Tukey’s multiple comparisons test. Statistical significance was denoted by asterisks: *p < 0.05, **p < 0.01, and ***p < 0.001.

## 3 Results

### 3.1 Berbamine exhibited significant anti-breast cancer activity

To evaluate the anti-tumor effects of BBM on breast cancer cells, MDA-MB-231, MDA-MB-468, MCF-7, and 4T1 cells were treated with BBM at different concentrations ranging from 0 to 80 μM for 48 h. CCK-8 assays revealed that BBM significantly inhibited the viability of these breast cancer cell lines in a dose- and time-dependent manner (*p* < 0.05). The IC_50_ values for MDA-MB-231, MDA-MB-468, MCF-7, and 4T1 cells were respectively 33.15 ± 7.08 μM, 31.47 ± 3.24 μM, 25.74 ± 5.59 μM, and 23.54 ± 3.08 μM (Figure 1A). In contrast, BBM exerted minimal cytotoxicity on normal mammary epithelial MCF10A cells, with an IC_50_ of 145.69 ± 7.87 μM (Figure 1B). Moreover, wound healing and Transwell invasion assays revealed that BBM markedly suppressed the migratory and invasive capacities of breast cancer cells (Figures 1C–F, *p* < 0.05). Collectively, these results indicate that BBM not only inhibits cell proliferation, migration, and invasion but also exhibits potent anti-breast cancer activity with low toxicity toward normal breast epithelial cells. Thus, BBM represents a promising anti-breast cancer agent.

**FIGURE 1:**
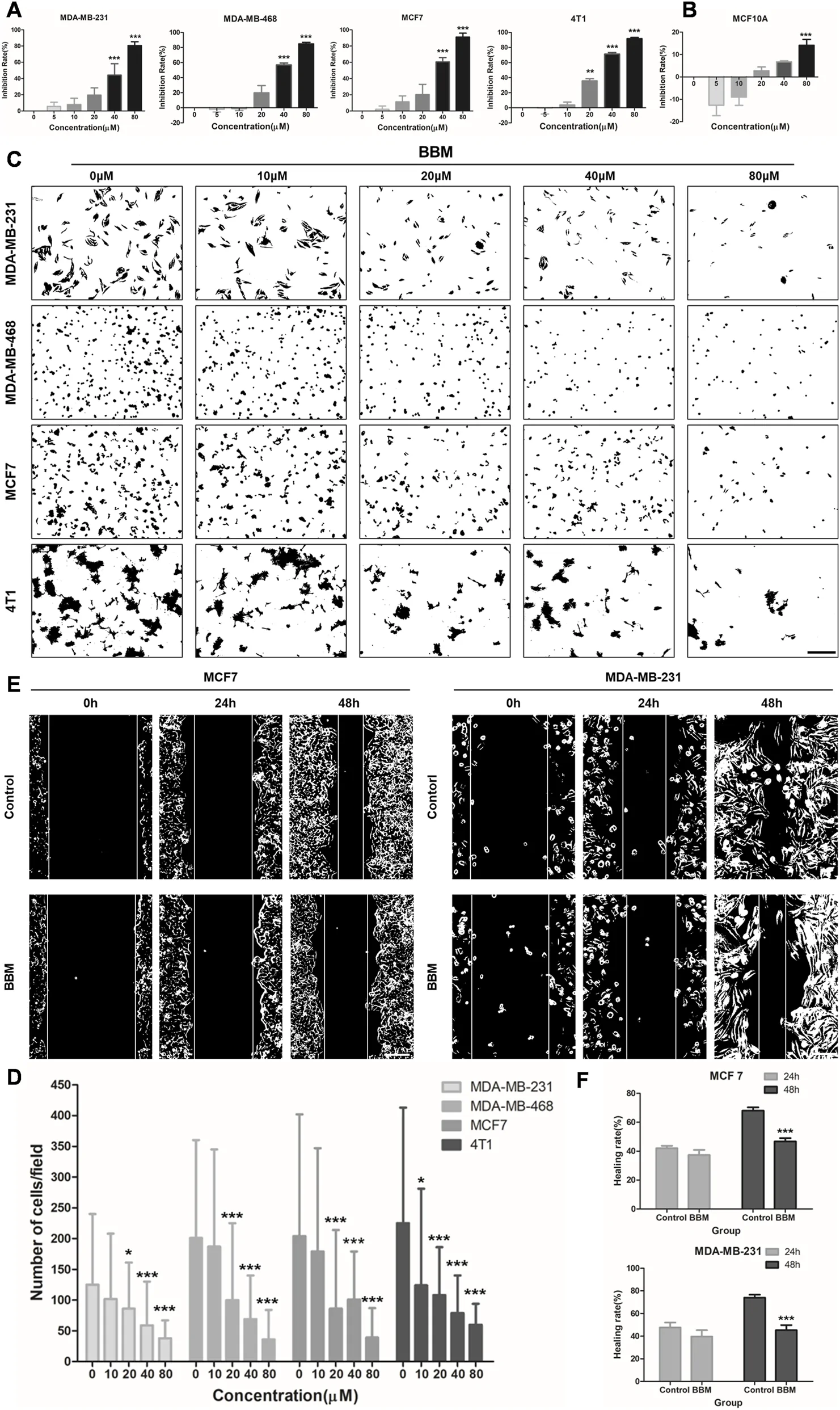
Berbamine inhibited the proliferation, migration, and invasion of breast cancer cells. **(A)** CCK-8 assay showing dose-dependent inhibitory effects of BBM (0–80 μM, 48 h) on the viability of breast cancer cell lines MDA-MB-231, MDA-MB-468, MCF-7, and 4T1. **(B)** BBM exhibited lower cytotoxicity in normal human breast epithelial MCF10A cells compared to cancer cell lines. **(C,D)** Transwell invasion assays showing the reducedinvasive capability of MDA-MB-231 and MCF-7 cells treated with BBM (25 μM) after 48 h. Scale bars: 100 μm. **(E,F)** Wound healing assays demonstrating the suppressive effect of BBM (0–80 μM) on the migration of MDA-MB-231 and MCF-7 cells after 24 and 48 h. Scale bars: 200 μm. Data are presented as mean ± SEM; *n* = 3; *p < 0.05, **p < 0.01, ***p < 0.001 versus 0 μM.

### 3.2 BBM induced autophagy-associated changes in breast cancer cells

Based on the preliminary inhibitory rate results obtained from the 4 cell lines (MCF-7, MDA-MB-231, MDA-MB-468, and 4T1) and considering their distinct molecular characteristics, MCF-7 (luminal A, non-invasive) and MDA-MB-231 (triple-negative, highly invasive) were selected for subsequent mechanistic studies on autophagy. Compared with the 0 h group, BBM treatment significantly increased the levels of p-ULK1, ATG5, ATG7, and Beclin1 in MCF-7 and MDA-MB-231 cells (Figures 2A–D, *p* < 0.05). Interestingly, p62 expression initially decreased and then gradually increased after BBM exposure, suggesting dynamic changes in autophagy-related processing (Figures 2A–D). To further validate autophagy induction, the eGFP-mRFP-LC3 adenoviral construct was used to monitor autophagosome formation. The number of LC3 puncta markedly increased after BBM treatment in both MCF-7 and MDA-MB-231 cells, indicating autophagosome accumulation (Figures 2E–H, *p* < 0.05). In addition, transmission electron microscopy (TEM) analysis confirmed the presence of numerous double-membraned autophagosomes in BBM-treated cells (Figure 2I). These findings demonstrated that BBM significantly activated autophagy in breast cancer cells.

**FIGURE 2:**
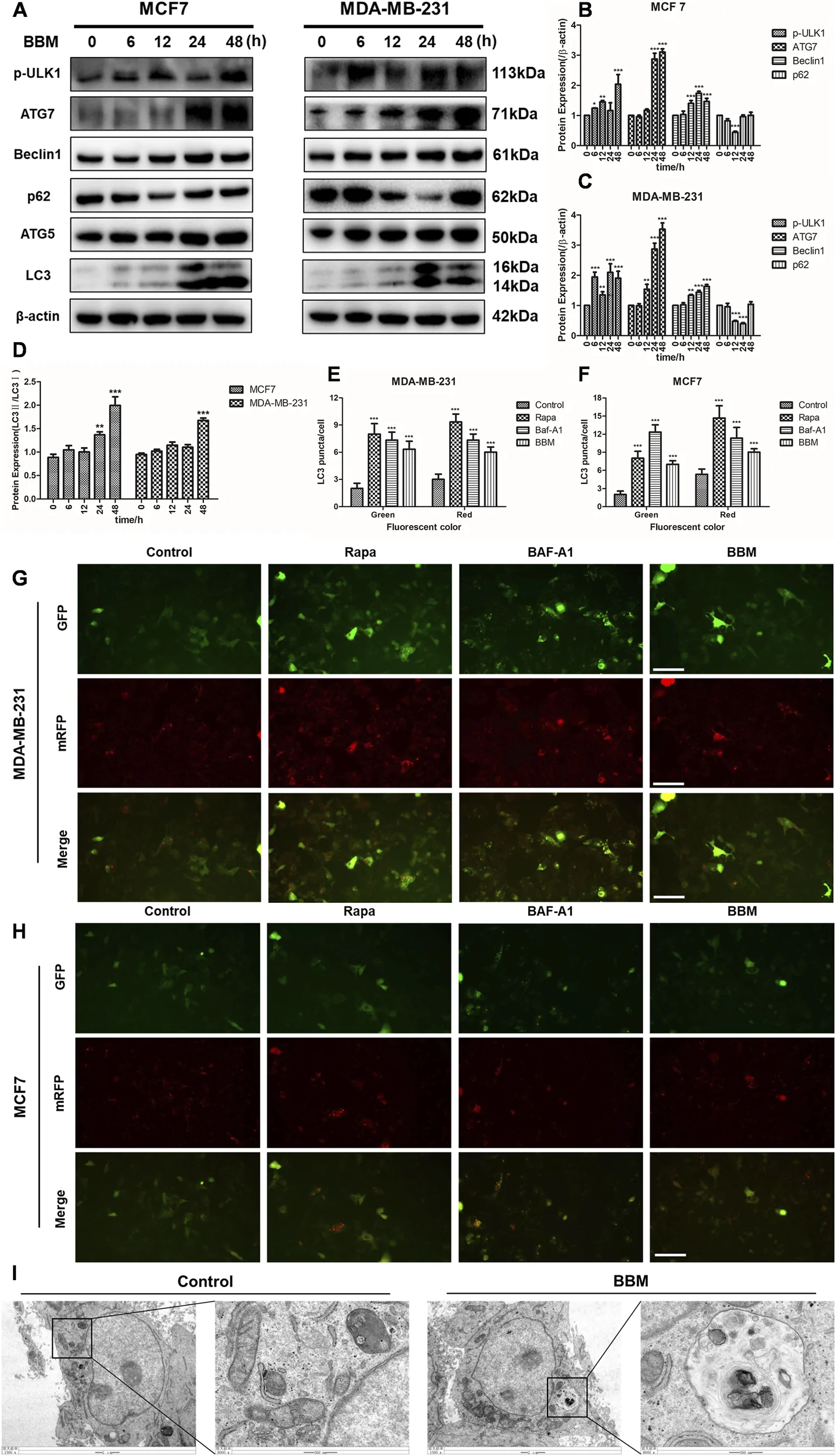
BBM induced autophagy-associated changes in breast cancer cells. **(A–D)** Western blot analysis of the expression of autophagy-related proteins (p-ULK1, ATG5, ATG7, p62, and Beclin1) in MCF-7 and MDA-MB-231 cells treated with BBM (25 μM). **(E–H)** Fluorescence imaging of LC3 puncta in MCF-7 and MDA-MB-231 cells infected with mRFP-GFP-LC3 adenovirus and treated with BBM (25 μM). Scale bars: 100 μm. **(I)** TEM images showing the accumulation of double-membraned autophagosomes in BBM-treated MDA-MB-231 cells. Scale bars: 2 μm. Data are expressed as mean ± SEM; *n* = 3; *p < 0.05, **p < 0.01, ***p < 0.001 versus 0 h.

To determine whether autophagy functionally contributes to the anticancer effects of BBM, MCF-7 and MDA-MB-231 cells were co-treated with BBM and either the autophagy activator rapamycin or the autophagy inhibitor Baf-A1. Rapamycin significantly enhanced the inhibitory effect of BBM on cell viability in both cell lines (Figure 3A), whereas Baf-A1 significantly attenuated the BBM-mediated reduction in cell viability (Figure 3B). Consistently, co-treatment with rapamycin markedly increased the expression of cleaved caspase-3 compared with BBM treatment alone, while Baf-A1 reduced cleaved caspase-3 levels relative to the BBM group (Figures 3C,D). These findings indicate that BBM induces autophagy-associated changes and promotes the accumulation of autophagosomes in breast cancer cells.

**FIGURE 3:**
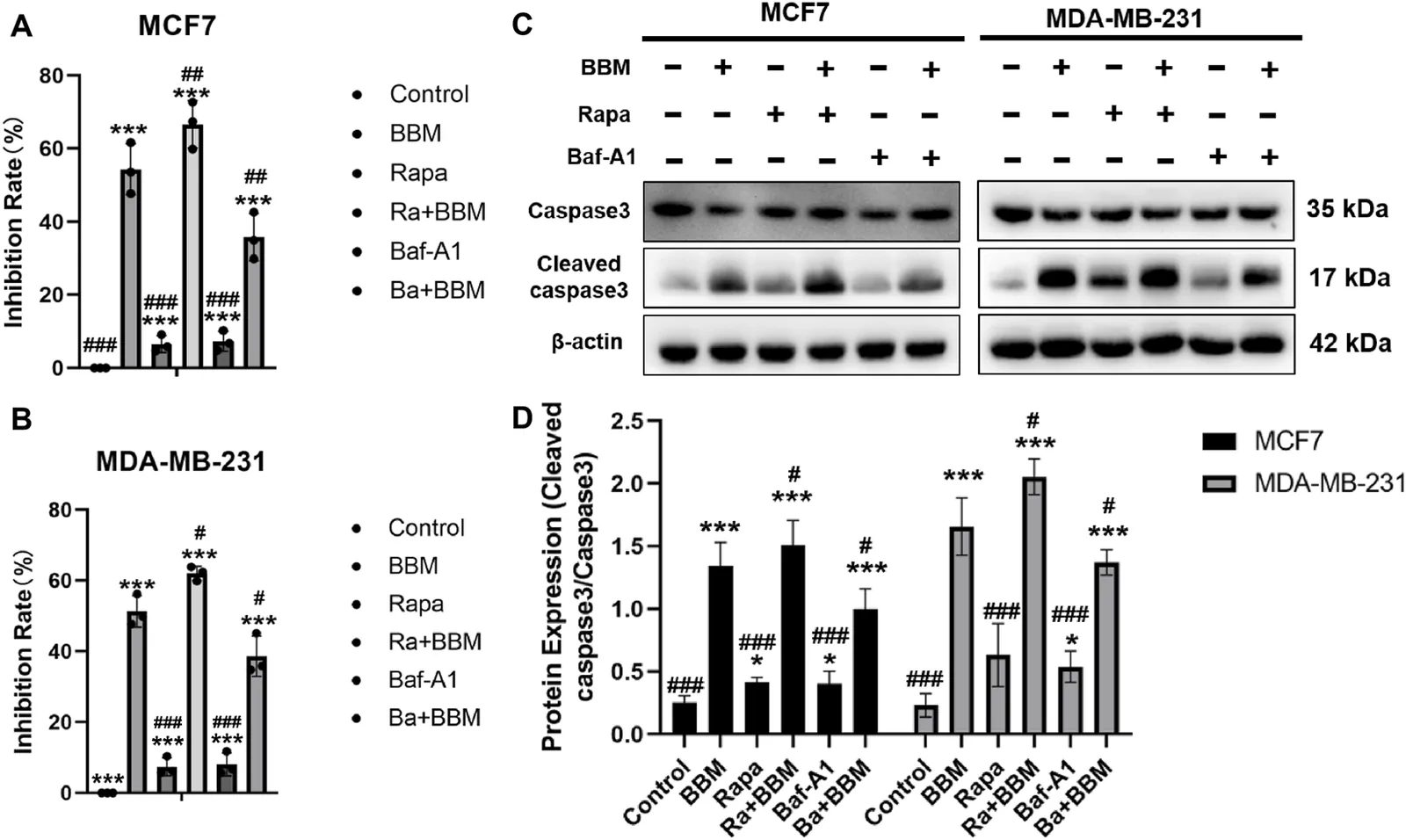
Autophagy modulation affected BBM-induced cytotoxicity in breast cancer cells. **(A)** Effects of rapamycin on the inhibitory effect of BBM on the viability of MCF-7 and MDA-MB-231 cells. **(B)** Effects of Baf-A1 on the inhibitory effect of BBM on cell viability. **(C,D)** Western blot analysis and quantification of cleaved caspase-3 expression in MCF-7 and MDA-MB-231 cells treated with BBM alone or in combination with rapamycin or Baf-A1. Data are expressed as mean ± SEM; n = 3; *p < 0.05, **p < 0.01, ***p < 0.001 versus the Control group; ^#^ *p* < 0.05, ^##^ *p* < 0.01, ^###^ *p* < 0.001 versus the BBM group.

### 3.3 BBM induced lysosome-associated responses in breast cancer cells

Lysosomal acidification and activity played critical roles in the fusion and degradation of autophagosomes, which were essential for autophagic flux. Numerous studies emphasized the necessity of intact lysosomal function for proper autophagy (Kendall and Holian, 2021; Medina, 2021). To examine lysosomal function, immunofluorescence staining and LysoTracker Red DND-99 labeling were employed. The results showed that BBM significantly promoted the nuclear translocation of TFEB, a key regulator of lysosomal biogenesis, in MDA-MB-231, MDA-MB-468, MCF-7, and 4T1 cells (Figures 4A–D). In addition, BBM increased the expression of lysosome-associated membrane protein 2 (LAMP2) in MDA-MB-231 and MCF-7 cells (Figure 4E, *p* < 0.05) and increased LysoTracker-positive acidic compartments (Figure 4F). These findings indicate that BBM treatment is associated with increased lysosome-related responses in breast cancer cells.

**FIGURE 4:**
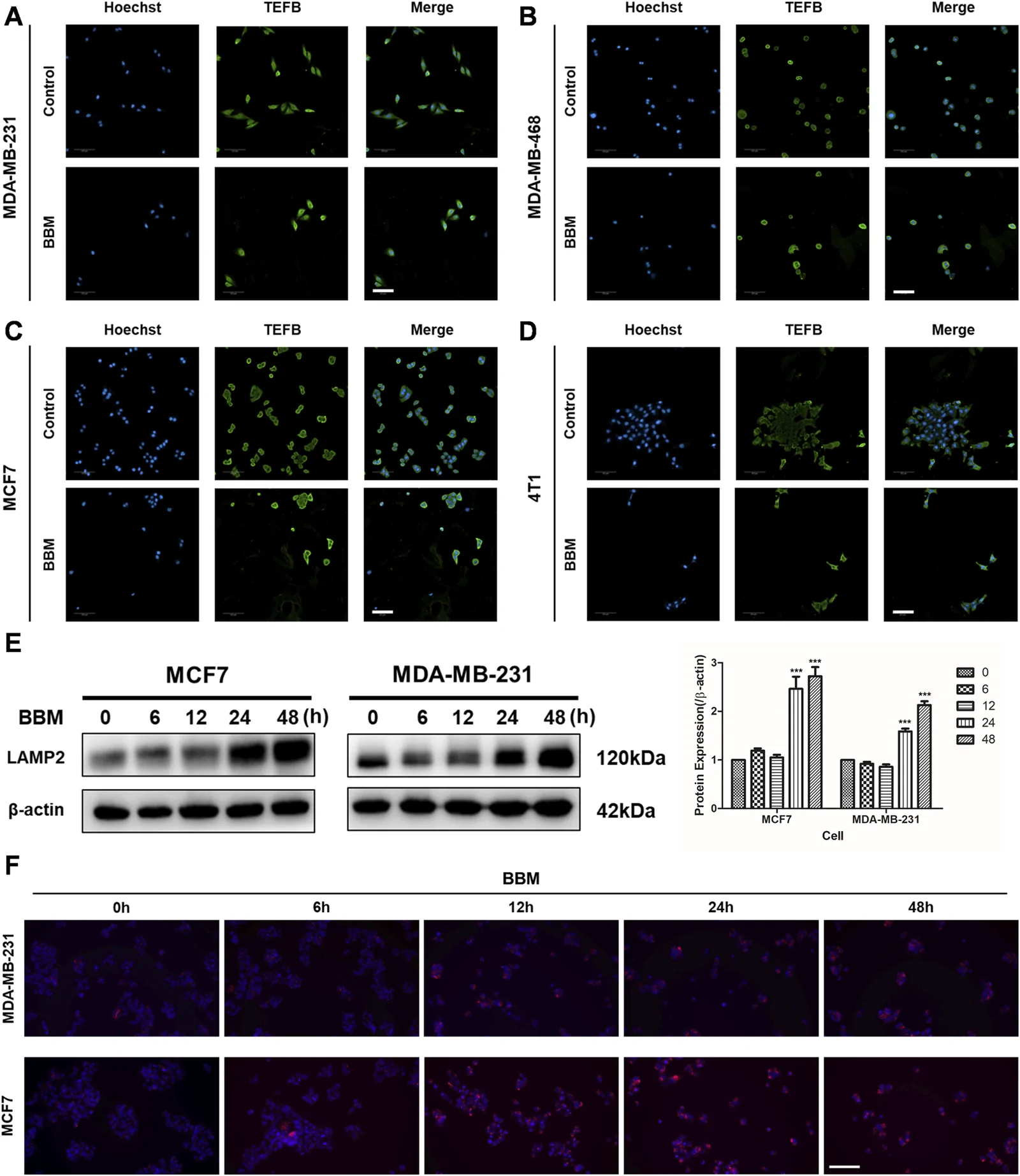
BBM was associated with lysosome-related responses in breast cancer cells. **(A–D)** Immunofluorescence staining of TFEB in MDA-MB-231, MDA-MB-468, MCF-7, and 4T1 cells treated with BBM (25 μM) after 48 h, demonstrating the increased nuclear translocation of TFEB. Scale bars: 100 μm. **(E)** Western blot analysis demonstrating that BBM (25 μM) significantly increased LAMP2 expression in MCF-7 and MDA-MB-231 cells. **(F)** LysoTracker Red staining indicating the increased LysoTracker-positive acidic compartments in MDA-MB-231 and MCF-7 cells after BBM treatment. Scale bars: 100 μm. All data are presented as mean ± SEM; *p < 0.05 versus control.

### 3.4 Exploratory network analysis identifies candidate BBM-associated targets and pathways

Based on the two GEO datasets (GSE42568 and GSE124646), we first identified differentially expressed genes (DEGs) between breast cancer and normal breast tissues (Figures 5A,B). Using an adjusted p-value <0.05 and an absolute log2 fold-change ≥1, the union of the DEGs from the two datasets contained 10,534 genes. Concurrently, 106 putative BBM targets were predicted based on the chemical structure of BBM (Figure 5C). The Venn diagram indicated that 34 targets overlapped between GSE42568 and the predicted BBM targets, 7 targets overlapped between GSE124646 and the predicted BBM targets, and 14 targets were shared by all three datasets (Figure 5D). Thus, 55 candidate BBM-associated overlapping targets were identified in total. The relationships among BBM, breast cancer, and these candidate targets were visualized using a drug–target–disease network (Figure 5E).

**FIGURE 5:**
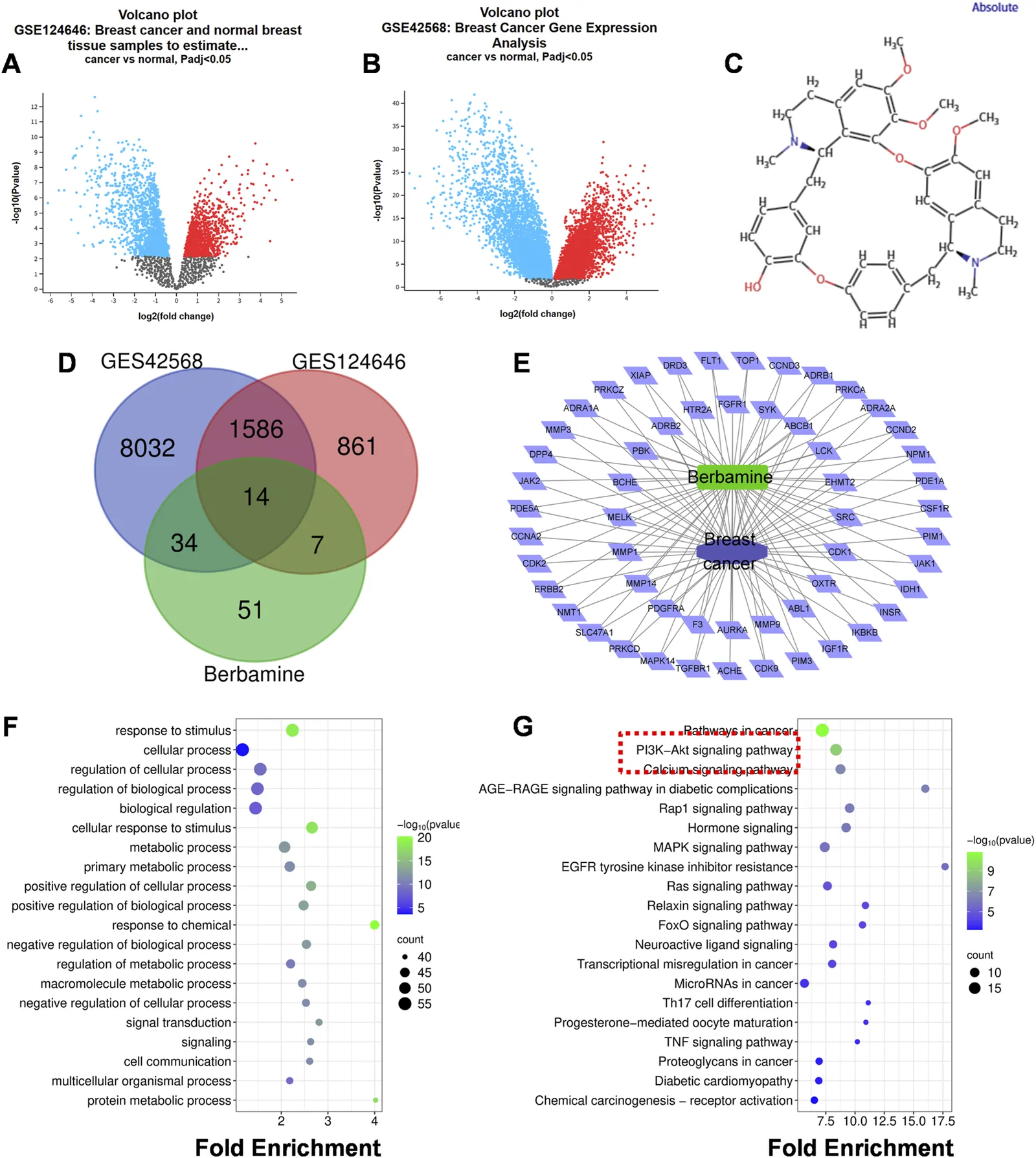
Exploratory network analysis of candidate BBM-associated targets and pathways. **(A,B)** Volcano plots showing differentially expressed genes (DEGs) between breast cancer and normal tissues in the GSE124646 **(A)** and GSE42568 **(B)** datasets (adjusted *p* < 0.05). Red dots represent upregulated genes, blue dots represent downregulated genes, and gray dots indicate non-significant genes. **(C)** Chemical structure of berbamine, reproduced from the PubChem Compound Database (CID 275182; National Center for Biotechnology Information: https://pubchem.ncbi.nlm.nih.gov/compound/275182). **(D)** Venn diagram illustrating the intersection between breast cancer-related DEGs and predicted berbamine targets. **(E)** Drug-target-disease network constructed using Cytoscape (version 3.9.1). **(F)** Bubble plot showing the top 20 Gene Ontology (GO) biological process (BP) terms enriched among the 55 candidate BBM-associated targets, ranked by gene count. **(G)** Bubble plot showing the top 20 Kyoto Encyclopedia of Genes and Genomes (KEGG) pathways enriched among the 55 candidate BBM-associated targets, ranked by gene count.

The 55 candidate targets were subjected to exploratory enrichment analysis, and the top 20 pathways ranked by gene count were visualized (Figures 5F,G). The enriched pathways included PI3K-Akt, MAPK, Rap1, FoxO, Ras, calcium, and AGE-RAGE signaling, among others. These findings identified several candidate pathways for further investigation and provided a rationale for subsequent experimental evaluation of the PI3K/Akt1/mTOR pathway.

### 3.5 BBM-associated autophagy changes and PI3K/Akt1/mTOR pathway modulation

To examine the involvement of PI3K/Akt1/mTOR signaling in the autophagy-related effects of BBM, we prioritized this pathway based on its established role in autophagy and breast cancer, previous reports linking this pathway to BBM responses, and the exploratory network analysis (Singh et al., 2017). Molecular docking predicted potential interactions between BBM and PI3K, Akt1, and mTOR (Figure 6A). Western blot analysis showed that BBM treatment reduced the phosphorylation levels of PI3K, Akt1, and mTOR in MCF-7 and MDA-MB-231 cells without substantially changing their total protein levels (Figures 6B–D). These findings support the involvement of PI3K/Akt1/mTOR pathway modulation in BBM-associated autophagy-related responses. Together with the pharmacological modulation experiments, the results support a functional contribution of autophagy-related processes to BBM-induced cytotoxicity.

**FIGURE 6:**
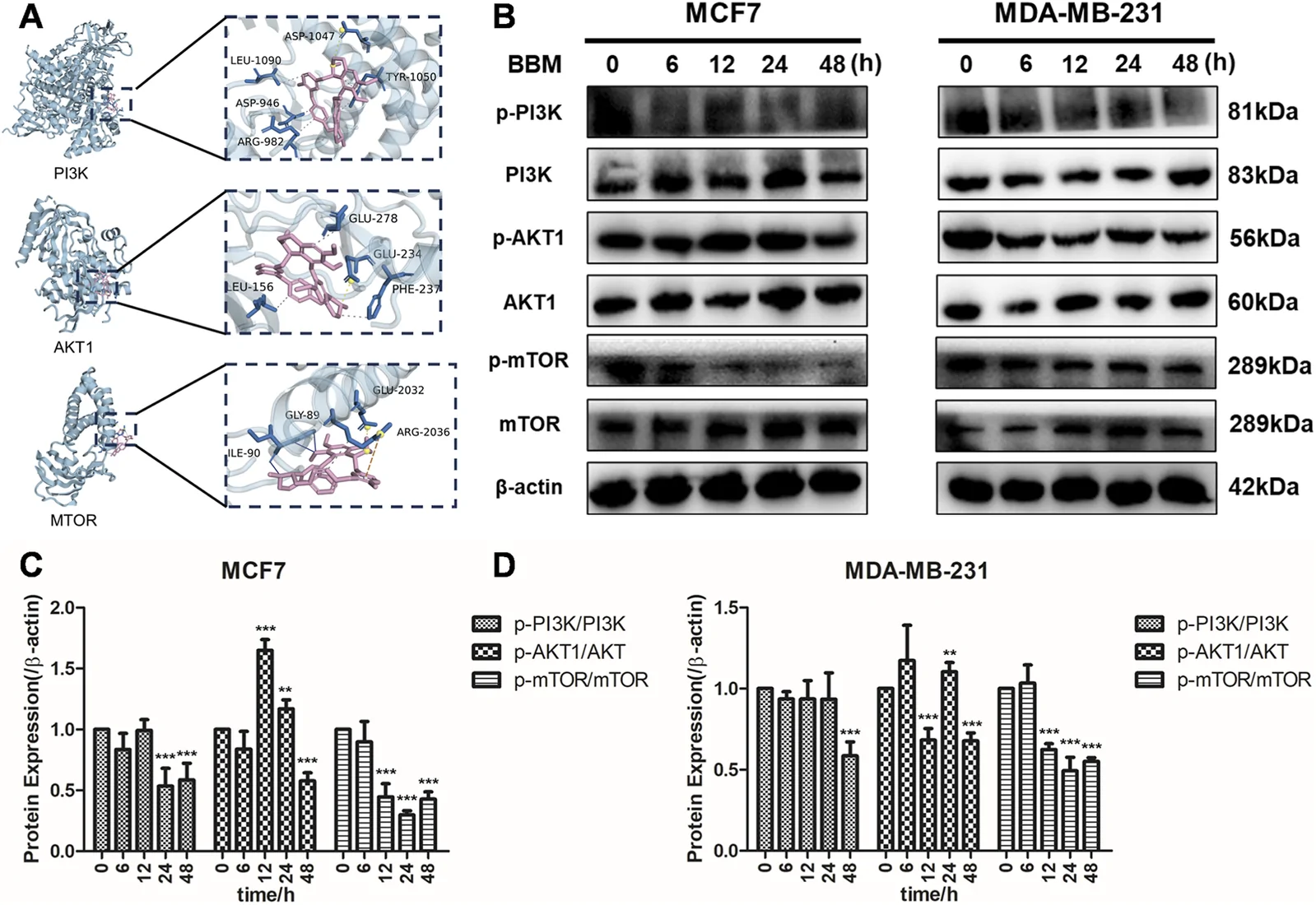
BBM-associated autophagy changes and PI3K/Akt1/mTOR pathway modulation. **(A)** Molecular docking results showing predicted binding poses of BBM with PI3K, Akt1, and mTOR. **(B–D)** Western blot analysis demonstrating that BBM (25 μM) significantly reduced the phosphorylation levels of PI3K, Akt1, and mTOR in MCF-7 and MDA-MB-231 cells, while total protein levels remained unchanged. Data are expressed as mean ± SEM; *n* = 3, *p < 0.05, **p < 0.01, ***p < 0.001 versus 0 h.

### 3.6 BBM inhibited xenograft growth and was associated with autophagy-related changes

Autophagy is a dynamic cellular process that regulates tumor cell homeostasis and treatment responses (Yu et al., 2024). Our previous *in vitro* experiments showed that BBM treatment was associated with autophagy-related changes and cytotoxicity in breast cancer cells. To further validate these findings *in vivo*, we established a xenograft model by subcutaneously implanting MCF-7 cells into nude mice and administering BBM for 14 consecutive days. The results showed that BBM improved the general condition of mice. BBM-treated mice exhibited higher body weight compared to controls (Figure 7A). Moreover, BBM markedly inhibited the growth of breast cancer xenografts, as evidenced by reduced tumor volume and weight (Figures 7B–D, *p* < 0.05). Histological analysis based on H&E staining revealed that tumors in the control group were composed of densely packed cells, whereas BBM treatment induced the formation of intracellular vacuoles, particularly at tumor margins (Figure 7E). To characterize the autophagy-related molecular changes associated with BBM treatment, we examined key autophagy-related markers. BBM significantly suppressed the activation of phosphorylated mTOR (p-mTOR), upregulated the expressions of autophagy-associated proteins (ATG5, ATG7, and LC3-II), and downregulated p62 expression in tumor tissues (Figures 7F,G, *p* < 0.05). Immunohistochemical analyses further showed changes in the expression of Beclin1, LAMP2, ATG5, ATG7, LC3, and p62 in tumor tissues (Figures 7H,I). These findings were supported by both immunohistochemistry and Western blot analyses and were consistent with activation of autophagy-associated responses *in vivo*. However, the present xenograft data do not distinguish whether autophagy directly mediates tumor cell death or occurs concurrently with other cell-death mechanisms.

**FIGURE 7:**
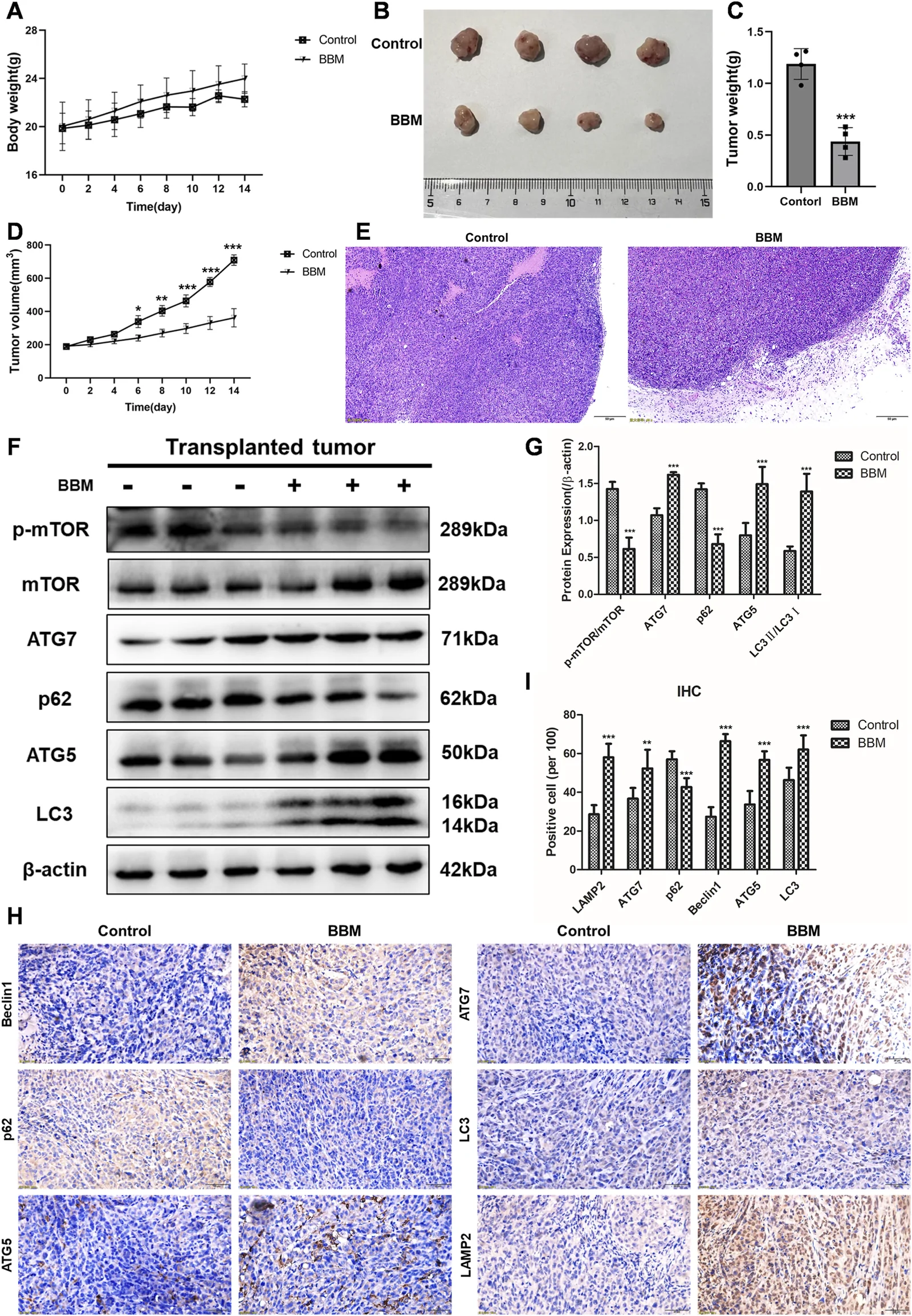
BBM inhibited xenograft growth and was associated with autophagy-related changes. **(A)** Body weight of nude mice in the control and BBM-treated groups after 14 days of treatment. **(B)** Representative photographs of excised xenograft tumors from nude mice in the control and BBM-treated groups at the end of the 14-day treatment period. **(C)** Tumor weight at the end of the 14-day treatment period. **(D)** Tumor volume measured every 2 days during treatment. **(E)** H&E staining of tumor tissues showing cellular morphology; BBM treatment led to the formation of intracellular vacuoles (black arrows). **(F,G)** Western blot analysis of p-mTOR, ATG5, ATG7, LC3-II, and p62 expressions in tumor tissues. **(H,I)** Immunohistochemical analysis of Beclin1, LAMP2, ATG5, ATG7, LC3, and p62 expressions in tumor tissues. Data are expressed as mean ± SEM; *n* = 4, *p < 0.05, **p < 0.01, ***p < 0.001 versus control group.

## 4 Discussion

In this study, we examined the anti-breast-cancer effects of BBM and the associated autophagy-, lysosome-, and signaling-related changes. BBM inhibited proliferation, migration, and invasion across multiple breast cancer cell lines while showing relatively low cytotoxicity toward normal mammary epithelial cells. BBM treatment was associated with autophagy-related marker changes, autophagosome accumulation, lysosome-related responses, and cancer cell cytotoxicity. Pharmacological modulation further supported a functional contribution of autophagy-related processes to BBM-induced cytotoxicity. These effects were accompanied by reduced phosphorylation within the PI3K/Akt1/mTOR pathway.

Our findings are consistent with the previously reported anti-proliferative, anti-migratory, and pro-apoptotic effects of BBM in breast cancer cells (Wang et al., 2009). In that study, BBM inhibited the growth, migration, and invasion of highly metastatic MDA-MB-231 breast cancer cells, induced apoptosis in MDA-MB-231 and MCF-7 cells, and showed relatively limited toxicity toward MCF-10A cells. Their findings also implicated inhibition of Akt-related signaling in the anti-cancer effects of BBM. The present study extends these findings by examining autophagy-associated responses, lysosome-related changes, and the effects of pharmacological autophagy modulation in MCF-7 and MDA-MB-231 cells. Instead, the present work extends the previous findings by examining autophagy-associated responses, lysosome-related changes, and the effects of pharmacological autophagy modulation in MCF-7 and MDA-MB-231 cells. In addition, our data associate BBM treatment with reduced PI3K/Akt1/mTOR phosphorylation, although the current docking and protein-expression data do not establish direct target engagement.

Autophagy is an evolutionarily conserved catabolic process that maintains cellular homeostasis through the degradation and recycling of damaged organelles and macromolecules (Miller and Thorburn, 2021; Russell and Guan, 2022). In cancer, autophagy plays dual roles, acting as a tumor-suppressive mechanism in early stages and a survival strategy in established tumors. Dysregulated or excessive autophagy leads to type II programmed cell death, also referred to as autophagic cell death (ACD) (Kroemer and Levine, 2008). In breast cancer, accumulating evidence suggests that autophagy modulation represents a promising therapeutic strategy. Agents that trigger ACD are particularly attractive for overcoming chemoresistance and eradicating aggressive tumor subtypes such as triple-negative breast cancer (TNBC) (Bhutta et al., 2024; Zhou et al., 2024).

The observed increase in lysosome-associated signals may reflect either a direct effect of BBM on lysosomal biogenesis and function or a compensatory response to the increased autophagic burden. BBM-induced accumulation of autophagic cargo could activate TFEB and the CLEAR network, thereby promoting lysosomal biogenesis and acidification as an adaptive response. Alternatively, BBM may directly affect lysosomal signaling or membrane-associated processes, resulting in enhanced lysosomal activity independently of autophagic cargo accumulation. The present findings, including TFEB nuclear translocation, increased LAMP2 expression, and enhanced LysoTracker staining, support an association between BBM treatment and lysosomal activation but do not establish whether this effect is direct or secondary to increased autophagic demand. Further studies using lysosomal proteolysis assays, lysosomal pH measurements, cathepsin activity assays, and autophagy-deficient models will be necessary to distinguish these possibilities.

Our findings indicated that BBM induced autophagy-associated changes in breast cancer cells, as evidenced by increased expression of classical autophagy-related markers (ATG5, ATG7, Beclin1, and p-ULK1), accumulation of LC3 puncta, and formation of double-membraned autophagosomes observed under transmission electron microscopy. Interestingly, p62 expression showed a biphasic pattern after BBM treatment, with an initial decrease followed by a late increase. The early decrease may reflect enhanced autophagic consumption of p62, whereas the later accumulation may result from an imbalance between autophagic cargo generation and lysosomal degradation. In particular, prolonged BBM exposure may lead to lysosomal saturation, incomplete autophagosome–lysosome fusion, or compensatory transcriptional upregulation of SQSTM1/p62. Because the present study did not directly distinguish among these possibilities, the late increase in p62 should not be interpreted solely as evidence of enhanced autophagic flux. Additional flux assays using Baf-A1 or chloroquine, together with SQSTM1/p62 transcript analysis, will be required to clarify the underlying mechanism (Zhan et al., 2022; Zhou et al., 2022). These findings are consistent with lysosomal activation; however, they do not establish whether lysosomal enhancement is a direct pharmacological effect of BBM or a compensatory response to the increased autophagic cargo generated by BBM treatment.

Because the present study did not directly measure lysosomal proteolytic activity, autophagosome-lysosome fusion, or lysosomal membrane integrity, the enhanced lysosomal markers should be interpreted cautiously. A previous study using MCF-7 and MDA-MB-231 cells reported that BBM inhibited late-stage autophagy by impairing autophagosome–lysosome fusion through the BNIP3–SNAP29–VAMP8 axis (Fu et al., 2018). In that study, BBM increased LC3-II and autophagosome accumulation, findings that were interpreted as impaired autophagic clearance rather than completion of autophagic flux. By contrast, our data showed induction-associated changes in p-ULK1, ATG5, ATG7, and Beclin1, together with TFEB nuclear translocation and increased LAMP2/LysoTracker signals, indicating enhanced autophagy initiation and lysosome-associated responses. Furthermore, rapamycin enhanced, whereas Baf-A1 attenuated, BBM-induced loss of cell viability and cleaved caspase-3 expression, supporting a functional contribution of autophagy-related processes to BBM-induced cytotoxicity. These apparently divergent observations may reflect differences in BBM exposure, treatment duration, cellular state, or experimental readouts. Alternatively, BBM may exert stage- and exposure-dependent effects, initially promoting autophagy initiation and lysosomal adaptation but subsequently causing autophagic cargo accumulation when fusion or degradation capacity becomes limiting. Because autophagosome–lysosome fusion and lysosomal proteolytic activity were not directly assessed in our study, a late-stage autophagy defect cannot be excluded. Therefore, our pharmacological rescue data support a functional contribution of autophagy to BBM-induced cytotoxicity, but they do not by themselves establish that BBM universally enhances complete autophagic flux or exclude a late-stage fusion defect.

We used exploratory network analysis to prioritize pathways for experimental investigation after observing the anti-breast-cancer effects of BBM. This analysis nominated PI3K-Akt signaling alongside MAPK, FoxO, and Rap1 signaling. We selected PI3K/Akt1/mTOR for further study because mTOR regulates autophagy through ULK1 (Kim et al., 2011), and previous work has associated BBM treatment with PI3K/Akt/mTOR signaling changes in breast cancer cells (Liu et al., 2021). In the present study, BBM treatment was accompanied by time-dependent changes in PI3K, Akt1, and mTOR phosphorylation in cultured cells, including reductions at later time points, and by reduced p-mTOR in MCF-7 xenograft tumors. Taken together, the prior evidence and our experimental observations support the involvement of this pathway in the BBM response under the conditions studied. The other enriched pathways remain directions for further investigation.

To validate the relevance of these findings *in vivo*, we established a breast cancer xenograft model using MCF-7 cells in BALB/c nude mice and administered BBM for 14 consecutive days. BBM-treated mice exhibited improved general conditions and maintained higher body weight compared with controls. More importantly, BBM significantly inhibited tumor growth, as reflected by reduced tumor volume and weight. Histological examination revealed pronounced cellular vacuolization in tumor tissues from BBM-treated mice, contrasting with the densely packed tumor cells observed in the control group. At the molecular level, BBM treatment was associated with reduced p-mTOR expression of phosphorylated mTOR (p-mTOR) and enhanced expression of autophagy-related proteins (ATG5, ATG7, and LC3-II) along with decreased p62 levels. These findings were supported by both immunohistochemistry and Western blot analyses and were consistent with activation of autophagy-associated responses *in vivo*; however, they do not by themselves establish definitive autophagic cell death.

It should be noted that the *in vivo* xenograft model used MCF-7 cells, which represent an estrogen receptor-positive luminal A subtype, whereas some of the strongest *in vitro* responses were observed in the triple-negative breast cancer cell lines MDA-MB-231 and 4T1. Therefore, the therapeutic efficacy and mechanism of BBM may differ across breast cancer subtypes. Accordingly, the antitumor efficacy observed in the MCF-7 xenograft model should not be directly extrapolated to TNBC. Previous studies have reported that BBM suppresses TNBC growth and motility through PI3K/Akt-related signaling (Wang et al., 2009; Liu et al., 2021), and autophagy-related signaling has been implicated in TNBC progression and treatment responses (Zhao et al., 2024; Zhou et al., 2024). Future studies using orthotopic or syngeneic 4T1 models, as well as TNBC patient-derived xenografts, will be important to determine whether BBM produces comparable or stronger antitumor effects in TNBC *in vivo*.

Our findings are consistent with previous reports highlighting the anticancer potential of BBM through modulation of the PI3K/Akt/mTOR pathway. Liu et al. reported that BBM suppressed the proliferation, migration, and invasion of triple-negative breast cancer cells through the PI3K/Akt/MDM2/p53 and PI3K/Akt/mTOR pathways (Liu et al., 2021). In liver cancer, BBM has also been reported to inhibit tumor growth by targeting Ca^2+^/calmodulin-dependent protein kinase II (Meng et al., 2013). Similarly, Mou et al. showed that BBM enhanced autophagic flux in colon cancer cells by targeting PI3K and modulating Beclin1 expression (Mou et al., 2019). These studies corroborate our observations and underscore the versatility of BBM as a modulator of autophagy across different cancer types. Furthermore, the selective cytotoxicity of BBM toward cancer cells over normal epithelial cells provides a significant therapeutic advantage. The IC_50_ values obtained from our CCK-8 assays indicate a clear therapeutic window, highlighting the potential of BBM as a relatively safe anticancer agent, an attribute particularly valuable given that dose-limiting toxicities often compromise outcomes with conventional chemotherapies.

Despite these compelling findings, several limitations warrant consideration. First, while our *in vitro* findings are supported by *in vivo* xenograft data, further exploration using orthotopic or patient-derived xenograft (PDX) models would better recapitulate the tumor microenvironment. Second, the rapamycin and Baf-A1 co-treatment experiments support a functional contribution of autophagy-related processes to BBM-induced cytotoxicity. However, these pharmacological agents may affect pathways beyond autophagy and therefore do not establish strict autophagy dependence. Moreover, autophagic flux, autophagosome–lysosome fusion, lysosomal proteolysis, and lysosomal membrane integrity were not directly quantified, and genetic manipulation of core autophagy genes will be needed to strengthen causal inference. Third, although autophagy appears to be a key mechanism underlying BBM-induced cell death, other pathways, such as apoptosis or ferroptosis, may also contribute and merit further investigation. Finally, the long-term impact of BBM on breast cancer stem-like cells and its potential to modulate the tumor microenvironment remain unexplored. The exploratory network analysis relies on database annotations and computational predictions and therefore requires further experimental validation. Direct target-engagement assays and genetic manipulation of relevant signaling and autophagy genes will be useful for strengthening the causal interpretation of the proposed mechanism. Addressing these questions in future studies will help establish a more comprehensive understanding of BBM’s therapeutic potential in breast cancer.

## 5 Conclusion

This study showed that BBM exerted anti-tumor effects against breast cancer cells *in vitro* and in an MCF-7 xenograft model. BBM inhibited the proliferation, migration, and invasion of breast cancer cells and showed relatively low cytotoxicity toward normal mammary epithelial cells. BBM treatment was accompanied by autophagy-related marker changes, autophagosome accumulation, lysosome-associated responses, and reduced phosphorylation within the PI3K/Akt1/mTOR pathway. The exploratory network analysis provided candidate molecular directions, while the *in vitro* and *in vivo* findings supported the involvement of PI3K/Akt1/mTOR signaling in BBM-associated responses. These findings support further investigation of BBM as a modulator of autophagy-related and PI3K/Akt1/mTOR-related processes in breast cancer.

## Data Availability

The original contributions presented in the study are included in the article/supplementary material, further inquiries can be directed to the corresponding authors.
